# Aberrant Expression of *TLR2*, *TLR7*, *TLR9*, Splicing Variants of *TLR4* and *MYD88* in Chronic Lymphocytic Leukemia Patients

**DOI:** 10.3390/jcm10040867

**Published:** 2021-02-19

**Authors:** Katarzyna Skorka, Paulina Wlasiuk, Agnieszka Karczmarczyk, Krzysztof Giannopoulos

**Affiliations:** Department of Experimental Haematooncology, Medical University of Lublin, 20-093 Lublin, Poland; katarzyna.skorka7@gmail.com (K.S.); paulina_wlasiuk@wp.pl (P.W.); agniecha_p86@o2.pl (A.K.)

**Keywords:** CLL (chronic lymphocytic leukemia), TLRs (toll-like receptors), Myd88 (myeloid differentiation primary response protein 88)

## Abstract

Functional toll-like receptors (TLRs) could modulate anti-tumor effects by activating inflammatory cytokines and the cytotoxic T-cells response. However, excessive TLR expression could promote tumor progression, since TLR-induced inflammation might stimulate cancer cells expansion into the microenvironment. Myd88 is involved in activation NF-κB through TLRs downstream signaling, hence in the current study we provided, for the first time, a complex characterization of expression of *TLR2*, *TLR4, TLR7*, *TLR9*, and *MYD88* as well as their splicing forms in two distinct compartments of the microenvironment of chronic lymphocytic leukemia (CLL): peripheral blood and bone marrow. We found correlations between *MYD88* and *TLRs* expressions in both compartments, indicating their relevant cooperation in CLL. The *MYD88* expression was higher in CLL patients compared to healthy volunteers (HVs) (0.1780 vs. 0.128, *p* < 0.0001). The TLRs expression was aberrant in CLL compared to HVs. Analysis of survival curves revealed a shorter time to first treatment in the group of patients with low level of *TLR4(3)* expression compared to high level of *TLR4(3)* expression in bone marrow (13 months vs. 48 months, *p* = 0.0207). We suggest that *TLRs* expression is differentially regulated in CLL but is similarly shared between two distinct compartments of the microenvironment.

## 1. Introduction

Chronic lymphocytic leukemia (CLL) is a disease with the accumulation of aberrant B cells in peripheral blood as well as proliferation and accumulation of CLL cells in the bone marrow and peripheral lymphoid organs. CLL patients are characterized by different prognoses, as well as profound molecular and immune defects [1,2,3]. Immune deregulations result in high susceptibility to infections as well as a failure to improve effective antitumor immune responses [4,5,6]. The nonspecific immune response could be continuously stimulated in CLL patients, although it has been suggested that the stimulation of toll-like receptors (TLRs) expressed on CLL cells could increase the immunogenicity of tumor cells and thus potentially contribute to the induction of tumor-specific immune response [7,8].

TLRs represent a family of transmembrane receptors that recognize a broad spectrum of pathogen-associated molecular patterns (PAMPs), such as highly conserved structures of viral (TLR7 or TLR9), bacterial (TLR2 or TLR4), and endogenous molecules released by injured tissues [8]. TLRs regulate innate immunity and determine the polarization and function of adaptive immunity mediated by B and T cells. TLRs expression is highly up-regulated through B-cell receptor (BCR) triggering of naive B-cells, indicating synergism between TLR and BCR leading to B-cell proliferation as well as differentiation [9]. All TLRs downstream signaling pathways except TLR3 are conducted by adaptor molecule Myd88 (myeloid differentiation primary response protein 88). It has been proven that TLRs types including 5, 7, 8, 9, 11 initiate the Myd88-dependent pathway directly. However, Myd88-dependent signaling pathway through TLRs types 1, 2, 4, 6 involves TIR—domain-containing adaptor protein (TIRAP). TLR3 and TLR4 initiate an alternative pathway which is MyD88-independent by recruiting TIR-domain-containing adaptor protein, inducing IFN-β (TRIF) [10]. Activation of MyD88 plays a crucial role in inflammatory cytokine secretion. The MyD88-dependent signaling pathway leads to the early phase of nuclear factor-κB (NF-κB) activation, whereas the MyD88-independent signaling pathway initiates the late phase of NF-κB activation. TLR-dependent signals could be also involved in the regulation of B lymphocytes function by inducing TLR tolerance or auto-reactivity promotion [11]. TLR7 and TLR9 engaged together with BCR also participate in the response to auto-antigens [12]. Recent studies indicated that both auto- and exogenous antigens might be engaged in the initiation and progression of CLL. The majority of data emphasized the role of adaptive immune receptors, including BCR, in the pathogenesis and progression of the disease [13,14,15].

The consequence of the occurrence of the *MYD88* mutation includes chronic activation of TLRs, thus the constitutive activation of the NF-κB promotes cell proliferation and survival [16]. Recent studies confirmed the prognostic value of *MYD88* mutation in CLL [17]. Since the *MYD88* mutation is harboring in 1–10% CLL patients, the current study aimed to characterize the association between expression and mutational status of *MYD88* and TLRs expression in the bone marrow and peripheral blood in CLL.

Splicing variants of TLRs might have different abilities to induce signal transduction since alternative splicing produces various transcripts and, as a result, various proteins from a single gene [18]. The current study aimed to present an expression pattern of *TLR2*, *TLR7*, *TLR9,* and splicing variants expression of *TLR4* (*TLR4(1), TLR4(3)*, *TLR4(4))* on the mRNA level as well as perform a comparison in two different microenvironmental compartments, peripheral blood and bone marrow, referring to recognized prognostic markers as well as clinical outcome.

## 2. Materials and Methods

### 2.1. Study Subjects

The material was obtained from 94 untreated CLL patients diagnosed at the Department of Hematooncology and Bone Marrow Transplantation, the Medical University of Lublin (60 males, 34 females, mean age 65). In the current study, we included patients with CLL diagnosed by aberrant immunophenotype, including CD5+ CD19+. The median value of CD19+ CD5+ expression analyzed on peripheral blood mononuclear cells (PBMC) was always >90%.

Thirty-three patients were classified in stage A, thirty-four in stages B, and eleven in stages C, according to the Binet classification. Detailed characteristics of CLL patients are shown in Table 1. This material was also obtained from 27 healthy volunteers. This study was approved by the Local Ethics Committee (KE-0254/7/2019), and the patients were informed about the use of their blood for scientific purposes.

### 2.2. Isolation of Mononuclear Cells

Mononuclear cells from peripheral blood (PBMC) and bone marrow (BMMC) were isolated by Ficoll (Biochrom AG, Berlin, Germany) density gradient. Next, cells were washed twice in phosphate-buffered saline (Biochrom AG, Berlin, Germany) and counted. The viability of obtained PBMC and BMMC was always >95%, as determined by trypan blue exclusion (Sigma-Aldrich, Schnelldorf, Germany). The viable cells were quantified in a Neubauer chamber (Zeiss, Jena, Germany) and stored for RNA preparation at −192 °C in liquid nitrogen.

### 2.3. RNA Isolation and Reverse Transcription

For the isolation of mRNA from PBMC, the QIAamp RNA Blood Mini Kit (Qiagen, Venlo, The Netherlands) was used according to the manufacturer’s instructions. One µg of mRNA was reverse transcribed into 20 μL of cDNA using a QuantiTect Reverse Transcription Kit (Qiagen, Venlo, The Netherlands). For each RT-PCR, 1 μL of the cDNA preparation was used.

### 2.4. Quantitative Polymerase Chain Reaction (q-PCR)

For the quantitative measurement of the mRNA expression of *TLR2*, *TLR4*, *TLR7* and *TLR9,* real-time q-PCR was performed using the Light Cycler SYBR Green I technology according to the manufacturer’s protocol (Roche Diagnostics, Rotkreuz, Switzerland). The sequences of the primers for q-PCR are shown in Table 2 (Roche Diagnostics, Rotkreuz, Switzerland).

The glyceraldehyde-3-phosphate dehydrogenase gene (GAPDH) was used as a housekeeping gene. An initial denaturation step at 95 °C for 10 min was followed by 40 cycles of 15 s at 95 °C and 1 min at 60 °C. The qPCR reactions were carried out using 7300 Real Time PCR System (Applied Biosystems, Foster City, CA, USA).

The *TLRs* mRNA expression was calculated as an inverse ratio of the difference in cycle threshold (ΔCt), where ΔCt is the Ct value of the target gene minus Ct value of *GAPDH*.

For the quantitative measurements of the mRNA expression of *MYD88,* qPCR was performed using TaqMan Universal PCR Master Mix and TaqMan Gene Expression Assay primer/probe mixes (Applied Biosystems, Foster City, CA, USA) according to the manufacturer’s instructions. *GAPDH* was used as a reference gene. Thermocycling program was set for 40 cycles of 15 s at 95 °C and 1 min at 60 °C on the 7300 Real-Time PCR System (Applied Biosystems, Foster City, CA, USA). The *MYD88* mRNA expression was calculated as an inverse ratio of the difference in cycle threshold (ΔCt), where ΔCt is the Ct value of the target gene minus Ct value of *GAPDH*.

### 2.5. Detection of the MYD88 L265P Mutation by the Allele Specific-Polymerase Chain Reaction (AS-PCR)

The *MYD88* L265P mutation was analyzed in 61 CLL patients in blood samples using the three primers: the mutant-specific reverse primer—5′-CCT TGT ACT TGA TGG GGA ACG-3′, the wild-type-specific reverse primer—5′-GCC TTG TAC TTG ATG GGG AAC A-3′, and the common forward primer—5′-AAT GTG TGC CAG GGG TAC TTA G-3′. Two reverse primers were designed to differentiate the mutant and wild-type allele of *MYD88* L265P before the PCR reaction DNA isolation was performed using QIAamp DNA Blood Mini Kit (Qiagen, Venlo, The Netherlands) according to the manufacturer’s instructions. PCR was performed in a total reaction volume of 25 μL with 10 pmol of each primer and 100 ng DNA using QIAGEN Multiplex PCR Kit (Qiagen, Hilden, Germany). Thermal cycling conditions were: 2 min of preheating at 94 °C followed by 40 cycles of 94 °C for 30 s, 57 °C for 30 s, and 68 °C for 30 s, with a final extension at 68 °C for 5 min. The PCR products (159 bp) were separated on 2% agarose gel electrophoresis and visualized under UV light.

### 2.6. Statistical Analysis

Statistical analysis was performed using GraphPad Prism 5. All results are presented as median values with rage. The U Mann–Whitney test and Kruskal–Wallis test was used to evaluate the difference between subgroups of patients. The correlations of variables were computed with Spearman’s rank correlation coefficient. Survival curves were calculated for time to first treatment (TTFT) of CLL patients according to the Kaplan–Meier method using a log-rank test. TTFT was calculated in months since the date of initial diagnosis until the date of initial treatment. For the subgroup of patients who had never received any treatment, we characterized TTFT as the date of the last follow up.

## 3. Results

### 3.1. Aberrant Expression of TLR2, TLR7, TLR9 and Splicing Variants of TLR4 in CLL Patients Compared to Healthy Volunteers

**Figure 1:**
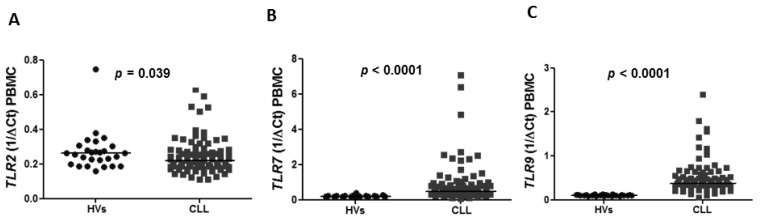
Expression of *TLR2*, *TLR7*, *TLR9* in CLL patients compared to healthy volunteers (HVs) in peripheral blood mononuclear cells (PBMC). (**A**) Lower expression of *TLR2* in PBMC in CLL patients compared to healthy volunteers (HVs) (0.2185 vs. 0.2632 (*p* = 0.039). (**B**) Higher expression of *TLR7* in PBMC in CLL patients compared to HVs (0.4790 vs. 0.1877, *p* < 0.0001). (**C**) Expression of *TLR9* in blood samples of CLL patients was significantly higher compared to HVs (0.3735 vs. 0.1066, *p* < 0.0001). Each dot (HV) or square (CLL) represents one individual case.

**Figure 2:**
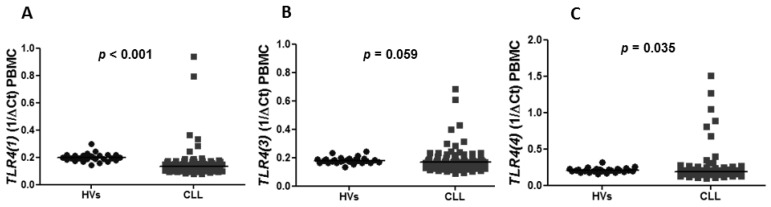
*TLR4* splice variants expression in CLL patients compared to healthy volunteers (HVs) in peripheral blood mononuclear cells (PBMC). (**A**) Lower expression of *TLR4(1)* in CLL patients than in HVs in PBMC (0.1330 vs. 0.1970, *p* < 0.0001). (**B**) No difference in expression of *TLR4(3)* in HV and CLL patients (0.1775 vs. 0.1680, *p* = 0.0592). (**C**) Lower expression of *TLR4(4)* in CLL patients compared to HVs (0.1840 vs. 0.2066, *p* = 0.0353). Each dot (HV) or square (CLL) represents one individual case.

The expression of *TLR2*, *TLR7*, *TLR9* and splicing variants of *TLR4* was confirmed in PBMC in CLL patients as well as in healthy volunteers (HVs). The expression of *TLR2* in peripheral blood was found to be lower in CLL patients compared to HVs with a median 0.2185 vs. 0.2632 (*p* = 0.039) (Figure 1A). Similarly, the expression of splicing variants of *TLR(4)*, including *TLR4(1)* and *TLR4(4),* was significantly lower in CLL patients than in HVs (median of *TLR4(1)*: 0.1330 vs. 0.1970, *p* < 0.0001 and median of *TLR4(4)*: 0.1840 vs. 0.2066, *p* = 0.0353) (Figure 2A,C). There was no difference in the expression of *TLR4(3)* in CLL patients and HVs (0.1680 vs. 0.1775, *p* = 0.0592) (Figure 2B). The expression of *TLR7* and *TLR9* was significantly higher in CLL patients compared to HVs (0.4790 vs. 0.1877, *p* < 0.0001), (0.3735 vs. 0.1066, *p* < 0.0001), respectively (Figure 1B,C).

### 3.2. Expression of TLR2, TLR7, TLR9 and Splicing Variants of TLR4 in Peripheral Blood and Bone Marrow Compartments in CLL

To analyze a difference in the regulation of *TLRs* expression in biologically various compartments, we performed a comparison of the levels of expression of TLRs in peripheral blood and bone marrow mononuclear cells. There was no difference in *TLR2*, *TLR7,* and *TLR9* expression in PBMC and BMMC with the median expression 0.2185 vs. 0.2 (*p* = 0.178); 0.479 vs. 0.4665 (*p* = 0.7215); 0.3735 vs. 0.368 (*p* = 0.8333), respectively. There was also no difference in expression of splicing variants of *TLR4* including *TLR4(1)*, *TLR4(3), TLR4(4)* in PBMC and BMMC (0.1330 vs. 0.1270, *p* = 0.8117), (0.1680 vs. 0.1520, *p* = 0.0952), (0.184 vs. 0.167, *p* = 0.0952).

### 3.3. Prognostic Value of the TLR2 Expression in Peripheral Blood and Bone Marrow Compartments in CLL

To characterize the significance of *TLRs* in CLL in a prognostic context we characterized the impact of *TLRs* expression on the clinical outcome and association with the recognized prognostic markers in both compartments, including PBMC and BMMC.

We observed no difference in TTFT in subgroups of patients with a high and low level of the *TLR2* expression in PBMC (*p* = 0.7229) as well as in BMMC (*p* = 0.4523).

**Figure 3:**
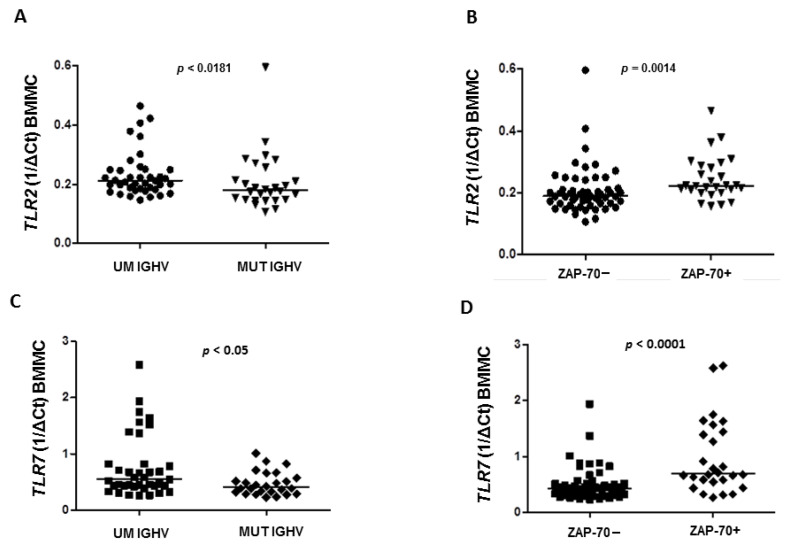
TLR2 and TLR7 expression in prognostically different CLL subgroups in refers to expression of ZAP-70 and mutational status of immunoglobulin heavy chain variable (IGHV) in bone marrow mononuclear cells (BMMC). (**A**) Higher expression of TLR2 in CLL patients with unmutated (UM) IGHV than in patients with IGHV mutation (MUT) (0.212 vs. 0.1795, *p* < 0.05). Each dot (UM IGHV) and triangle (MUT IGHV) represents an individual case. (**B**) Higher expression of TLR2 in ZAP-70+ patients compared to ZAP-70− (0.2225 vs. 0.1885, *p* = 0.0014). Each dot (ZAP-70+) and triangle (ZAP-70-) represents an individual case. (**C**) Higher expression of TLR7 in IGHV UM patients compared to IGHV MUT (0.549 vs. 0.407 *p* < 0.0361). Each square (UM IGHV) and rhombus (MUT IGHV) represents an individual case. (**D**) Higher expression of TLR7 in ZAP-70+ patients compared to ZAP-70− (0.694 vs. 0.424, *p* < 0.0001). Each square (ZAP-70-) and rhombus (ZAP-70+) represents an individual case.

We found that the expression of *TLR2* in BMMC was significantly higher in CLL patients with the unmutated status of the immunoglobulin heavy chain variable (UM *IGHV*) genes than in patients with *IGHV* mutation (MUT) with a median 0.212 vs. 0.1795 (*p* = 0.0181), respectively (Figure 3A).

The expression of *TLR2* in BMMC was confirmed to be higher in ZAP-70+ (defined as cytoplasmatic expression >20% CLL cells) patients compared to ZAP-70−, with a median 0.2225 vs. 0.1885 (*p* = 0.0014), respectively (Figure 3B). However, in PBMC there were no differences in the expression of *TLR2* depending on ZAP-70 expression (median expression 0.215 vs. 0.222, *p* = 0.8538) as well as the mutational status of *IGHV* genes (median expression 0.225 vs. 0.204, *p* = 0.8690).

We found that there was no difference in *TLR2* expression in PBMC (median expression 0.212 vs. 0.2185, *p* = 0.5737) and BMMC (median expression 0.2055 vs. 0.2, *p* = 0.9788) in the groups of patients defined by CD38 expression (cut-off value for the positive expression = 30%).

Additionally, there were no statistical differences in *TLR2* expression in stages A, B, and C according to Binet’s classification (Table S1). We did not find any correlation between expression of *TLR2* in PBMC and BMMC and CLL patient’s age (r = −0.04755, *p* = 0.6582), (r = 0.05174, *p* = 0.6301), respectively.

### 3.4. Prognostic Value of the TLR7 Expression in Peripheral Blood and Bone Marrow Compartments in CLL

Analysis of survival curves found no difference in TTFT in subgroups of patients with a high and low level of the *TLR7* expression in PBMC (*p* = 0.6819) as well as in BBMC (*p* = 0.5472).

In the groups of CLL patients categorized by the mutational status of *IGHV* genes and ZAP-70 expression, we found that in PBMC there were no differences in *TLR7* expression in *IGHV* MUT and *IGHV* UM CLL patients (0.4805 vs. 0.583, *p* > 0.05) as well as in ZAP-70− and ZAP-70+ CLL patients (0.4935 vs. 0.532, *p* = 0.3738). Interestingly, in BMMC we found that expression of *TLR7* was significantly higher in *IGHV* UM patients compared to *IGHV* MUT (0.549 vs. 0.407 vs., *p* < 0.0361) (Figure 3C) as well as in ZAP-70+ patients compared to ZAP-70− (0.694 vs. 0.424 vs., *p* < 0.0001) (Figure 3D).

There was no difference in *TLR7* expression in CD-38+ and CD-38− patients in both peripheral blood and bone marrow compartments (0.668 vs. 0.4805, *p* = 4515 and 0.6895 vs. 0.4435, *p* = 0.0647), respectively. No association was observed in *TLR7* expression in peripheral blood and bone marrow with the clinical stage of disease according to Binet’s classification (Table S1).

We did not find a correlation between the expression of *TLR7* in peripheral blood and bone marrow samples and CLL patient’s age (r = 0.1755, *p* = 0.1, r = 0.03711, *p* = 0.7299), respectively.

### 3.5. Prognostic Value of the TLR9 Expression in Peripheral Blood and Bone Marrow Compartments in CLL

We showed the tendency of shorter TTFT in groups of CLL patients with high *TLR9* expression in comparison to low *TLR9* expression in BBMC (12 vs. 45, *p* = 0.0655). In PBMC there was no difference in TTFT, referring to the level of *TLR9* expression (*p* = 0.3210).

There was no difference in the expression of *TLR9* in peripheral blood as well as in bone marrow in *IGHV* MUT CLL patients mutated compared to *IGHV* UM (0.4345 vs. 0.367, *p* = 0.2104; median expression 0.3375 vs. 0.353, *p* = 0.6380, respectively).

There was no difference in *TLR9* expression between groups of patients characterized by the expression of ZAP-70 both in peripheral blood (0.3524 vs. 0.4190, *p* = 0.2327) and bone marrow samples (0.3375 vs. 0.407, *p* = 0.1038), respectively. In PBMC we found that expression of *TLR9* was significantly higher in CD-38− compared to CD-38+ patients (0.4005 vs. 0.295, *p* = 0.0234). In contrast, in bone marrow samples we did not find a statistically significant difference in *TLR9* expression between CD-38+ and CD-38− patients (0.4025 vs. 0.3583, *p* = 0.6519).

No differences were observed in *TLR9* expression in peripheral blood between stages A, B, and C according to Binet’s classification in PBMC and BMMC (Table S1). We did not find any correlation between the expression of *TLR9* in peripheral blood and bone marrow samples and CLL patient‘s age (r = −0.01817, *p* = 0.8658: r = 0.07727, *p* = 0.4717), respectively.

### 3.6. Prognostic Value of the Expression of Splicing Variants of TLR4 (TLR4(1), TLR4(3), TLR4(4)) in Peripheral Blood and Bone Marrow Compartments in CLL

**Figure 4:**
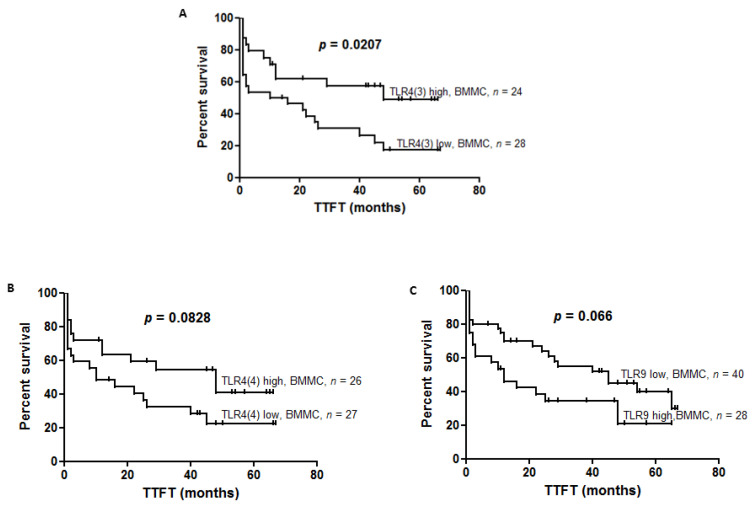
Time to first treatment (FFTF) in CLL patients divided according to the level of the *TLRs* expression in peripheral blood mononuclear cells (PBMC) and bone marrow mononuclear cells (BMMC). (**A**) TTFT was significantly shorter in the group of patients with a low level of *TLR4(3)* expression compared to the group of patients with a high level of *TLR4(3)* expression in BMMC (13 vs. 48, *p* = 0.0207). (**B**) TTFT tended to be shorter in the group of patients with a low level of *TLR4(4)* expression compared to the group of patients with a high level of *TLR4(4)* expression in BMMC (10 vs. 48, *p* = 0.0828). (**C**) TTFT tended to be shorter in the group of patients with a high level of *TLR9* expression compared to the group of patients with a low level of *TLR9* expression in BMMC (12 vs. 45, *p* = 0.0655).

To define the prognostic value of *TLR(4)* splicing variants, we divided TTFT according to the level of the *TLR4(1)*, *TLR4(3)*, *TLR4(4)* expression in PBMC and BMMC. Analysis of survival curves found that TTFT was not different when referring to the level of *TLR4(1)* expression in PBMC (*p* = 0.5674) as well as BMMC (*p* = 0.3008). We found shorter TTFT in patients with low expression of *TLR4(3)* compared to patients with high expression of *TLR4(3)* in BBMC (13 vs. 48, *p* = 0.0207) (Figure 4A). A tendency to have shorter TTFT was found in patients with low expression of *TLR4(4)* compared to high expression in BBMC (10 vs. 48, *p* = 0.0828) (Figure 4B). However, in PBMC we did not observe changes in TTFT regarding *TLR4(3)* (*p* = 0.01761) and *TLR4(4)* expression.

We analyzed the expression of splicing variants of *TLR4* (*TLR4(1)*, *TLR4(3)*, *TLR4(4)*) with respect to the mutational status of *IGHV* genes. There were no differences in expression of *TLR4(1)* in *IGHV* UM CLL cases compared to *IGHV* MUT (0.124 in vs. 0.141, *p* = 0.1316) in PBMC as well as in BMMC (0.1265 vs. 0.131, *p* = 0.493). We found that expression of *TLR4(3)* and *TLR(4)* in PBMC were lower in patients with *IGHV* UM compared to *IGHV* MUT (0.158 vs. 0.1815, *p* = 0.0233) (0.1695 vs. 0.2015 vs, *p* = 0.00642), respectively. The expression was found to be no different in BBMC in patients with *IGHV* MUT compared to *IGHV* UM (0.1585 vs. 0.137, *p* = 0.3188).

The expressions of *TLR4(1)*, *TLR4(3)* and *TLR4(4)* were analyzed in two groups of CLL patients: ZAP-70+ and ZAP-70−. The expression of *TLR4(1)* in PBMC was found to be lower in ZAP-70+ patients than in ZAP-70+ (median expression 0.122 vs. 0.139, *p* = 0.0305). In bone marrow samples we also did not find differences in expression of *TLR4(1)* in ZAP-70+ and ZAP-70− CLL patients (0.157 vs. 0.152, *p* = 0.7583). We evaluated the expression of *TLR4(3)* in those groups of CLL patients. We observed that there was no difference in expression of *TLR4(3)* in PBMC and BMMC in ZAP-70− and ZAP-70+ patients (0.172 vs. 0.159, *p* = 0.0541), (0.152 vs. 0.157, *p* = 0.7583), respectively. The expression of *TLR4(4)* was similar in ZAP-70− and ZAP-70+ patients (0.1965 vs. 0.173, *p* = 0.2039) in PBMC as well as in BMMC (0.17 vs. 0.168, *p* = 0.804).

We determined the expression of splicing variants of *TLR4 (TLR4(1), TLR4(3), TLR4(4)*) in CD38+ and CD38− CLL patients. There were no differences in expression of *TLR4(1)* (0.126 vs. 0.137, *p* = 0.3911), *TLR4(3)* (0.16 vs. 0.171, *p* = 0.3079) and *TLR4(4)* (0.192 vs. 0.184, *p* = 0.5018) in CD-38+ and CD-38− groups in PBMC. In bone marrow, differences in expression of *TLR4(1)* (0.121 vs.0.13 *p* = 0.4011), *TLR4(3)* (0.156 vs. 0.152, *p* = 0.6144), *TLR4(4)* (0.176 vs. 0.168, *p* = 0.679) in CD38+ and CD38− CLL patients were not also observed.

We did not find any difference in expression of splicing variants of TLR4 in stages A, B, and C according to Binet’s classification in PBMC and BMMC (Table S1). We did not find correlation between expression of splicing variants of *TLR4* in peripheral blood (*TLR4(1):* r = −0.0705, *p* = 0.5188, *TLR4(3)*: r = −0.1203, *p* = 0.2698, *TLR4(4)*: r = 0.8704) and bone marrow samples *TLR4(1)*:r = −0.1613, *p* = 0.1378, *TLR4(3)*: r = −0.04505, *p* = 0.6804, *TLR4(4)*: r = −0.02286, *p* = 0.8345) and CLL patient’s age.

### 3.7. Prognostic Value of the MYD88 Expression and the Association with TLRs Expression in CLL

**Figure 5:**
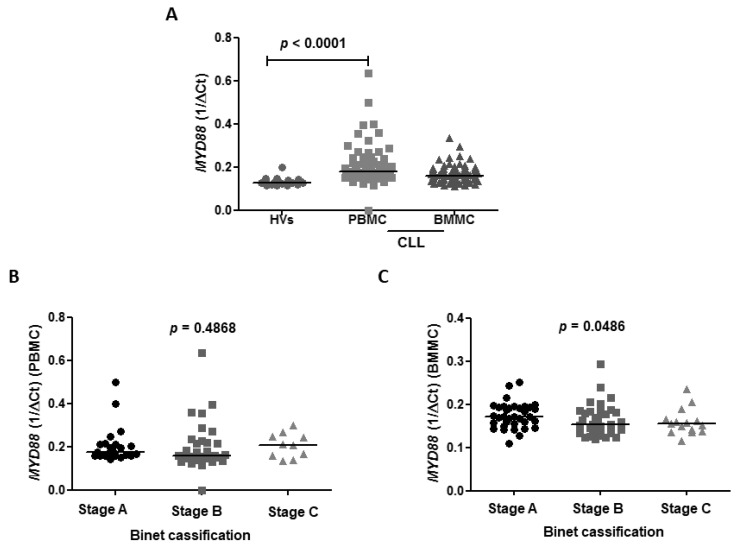
Expression of *MYD88* in peripheral blood mononuclear cells (PBMC) and bone marrow mononuclear cells (BMMC) of CLL compared to healthy volunteers (HVs) and association of *MYD88* expression with clinical stage of CLL according to Binet’s scale. (**A**) Higher expression of *MYD88* in CLL vs. HVs in PBMC (0.1780 vs. 0.1280, *p* < 0.0001). The median expression of *MYD88* in BMMC, *p* = 0.1600. Each dot (HV), square (PBMC) and triangle (BMMC) represent an individual case. (**B**) No association of expression of *MYD88* with clinical stage of CLL according to Binet’s scale in PBMC with the median expression in A, B, C Binet stages: 0.1765 vs. 0.1605 vs. 0.2070, *p* = 0.4868, respectively. Each dot (Stage A), square (Stage B) and triangle (Stage C) represents an individual case. (**C**) Higher expression of *MYD88* in A Binet stage compared to B and C Binet stage in BMMC (0.1720 vs. 0.1535 vs. 0.1550, *p* < 0.0001). Each dot (Stage A), square (Stage B) and triangle (Stage C) represents an individual case.

The *MYD88* expression was higher in CLL patients compared to HVs with a median of 0.1780 vs. 0.128 (*p* < 0.0001), respectively. The median expression of *MYD88* in BMMC was 0.1600 (Figure 5A). We revealed no differences in TTFT in subgroups of patients with high and low expression of *MYD88* in bone marrow (10 vs. 26, *p* = 0.92) as well as in peripheral blood (11 vs. 21, *p* = 0.59).

To identify association of *MYD88* expression and *TLRs* expression, we assessed correlations in blood and bone marrow samples (Table 3).

We showed a strong correlation between *MYD88* expression and *TLR2* in PBMC (r = 0.722, *p* ≤ 0.0001) and medium correlation in BMMC (r = 0.389, *p* = 0.0001) (Figure S1A,B). There were medium correlations between expression of *MYD88* and expression of *TLR4* splicing variants including *TLR4(1)* (r = 0.559, *p* < 0.001), *TLR(3)* (r = 0.558, *p* < 0.001), and *TLR4(4)* (r = 0.56, *p* < 0.001) in PBMC (Figure S2A,C,E). In BMMC there were weak associations between expression of *MYD88* and expression of *TLR4(1)* (r = 0.284, *p* < 0.007), *TLR(3)* (r = 0.294, *p* < 0.005), *TLR4(4)* (r = 0.44, *p* < 0.001) (Figure S2B,D,F). We observed medium correlation between *MYD88* expression and *TLR7* expression in PBMC (r = 0.580, *p* < 0.001) and medium correlation in BMMC (r = 0.358, *p* < 0.001) (Figure S3A,B) There were medium correlations between *MYD88* expression and *TLR9* expression in PBMC (r = 0.492, *p* < 0.001) as well as in BMMC (r = 0.541, *p* < 0.001) (Figure S4A,B).

To determine the significance of *MYD88* in a different compartment we performed statistical analyses referring to CLL subgroups of patients with different prognoses in blood as well as bone marrow samples. We observed no association of expression of *MYD88* with clinical stage of CLL according to Binet’s scale in PBMC with the median expression in A, B, C Binet’s stage: 0.1765 vs. 0.1605 vs. 0.2070, *p* = 0.4868, respectively (Figure 5B). However, BMMC expression of *MYD88* was found to be higher in A Binet stage compared to B and C Binet stages in BMMC (0.1720 vs. 0.1535 vs. 0.1550, *p* < 0.0001) (Figure 5C).

No correlation between *MYD88* expression in blood and bone marrow samples in CLL patients (r = 0.04, *p* = 0.715) was found.

We analyzed if the expression of *MYD88* in blood and bone marrow samples in CLL patients depends on the prognostic markers including the mutational status of *IGHV* genes, ZAP-70 expression, CD38 expression, and Binet stage. We observed no differences in the expression of *MYD88* in *IGHV* MUT patients compared to *IGHV* UM in PBMC with a median 0.1831 vs. 0.1926 (*p* = 0.4934), respectively, as well as in BMMC with a median 0.1603 vs. 0.1605 (*p* = 0.8730), respectively. There were no differences in the expression of *MYD88* in ZAP-70+ CLL patients compared to ZAP-70− with a median 0.1734 vs. 0.1816 (*p* = 0.6426) in PBMC and BMMC with a median 0.1646 vs. 0.1601 (*p* = 0.9589). There were also no differences in the expression of *MYD88* in CD38+ CLL patients compared to CD38− in PBMC with a median 0.2112 vs. 0.1778 (*p* = 0.867), respectively, as well as in BMMC with a median 0.1553 vs.0.1636 (*p* = 0.1855), respectively.

There were no correlations between *MYD88* expression and the age of CLL patients in peripheral blood (*p* = 0.7874) and bone marrow (*p* = 0.3189) samples. We did not find any associations between *MYD88* expression and morphological parameters, including the level of white blood cells (WBC) (*p* = 0.7873), red blood cells (RBC) (*p* = 0.537), platelets (PLT) (*p* = 0.2014), neutrophils (*p* = 0.981), and the level of hemoglobin (Hgb) (*p* = 0.570) and hematocrit (Hct) (*p* = 0.5017) in blood samples. There were no correlations between *MYD88* expression and morphological parameters, including the level of white blood cells (WBC) (*p* = 0.9314), red blood cells (RBC) (*p* = 0.1792), platelets (PLT) (*p* = 0.2209), neutrophils (0.1007), and the level of hemoglobin (Hgb) (*p* = 0.3833) and hematocrit (Hct) (*p* = 0.3389) in bone marrow.

### 3.8. MYD88 L265P Mutation in CLL Patients

*MYD88* L265P mutation occurred in 2/61 (3.28%) CLL patients in PBMC. Since a small number of the cohort had a *MYD88* mutation, we did not aim to obtain median and statistical data. We obtained mean values. In patients with *MYD88* L265P mutation, the mean expression of splicing variants of *TLR4* (1/ΔCt) was as follows: *TLR4(1)*—0.165, *TLR4(3)*—0.153, *TLR4(4)*—0.138. In the group of patients without *MYD88* L265P mutation, the median expression of splicing variants of *TLR4* (1/ΔCt) was 0.138 for *TLR4(1)*, 0.171 for *TLR4(3)*, and 0.184 for *TLR4(4)*. In the population of *MYD88*-mutated patients, the mean expression of *TLR2* (1/ΔCt) was 0.203, meanwhile patients without *MYD88* mutation had a median of 0.225. The mean expression of *TLR7* (1/ΔCt) in patients with *MYD88* L265P mutation was 0.866, and in the case of patients without *MYD88* L265P mutation, the median expression of *TLR7* (1/ΔCt) was 0.573. In patients harboring the *MYD88* L265P mutation, the mean expression of *TLR9* (1/ΔCt) was 0.517, meanwhile patients without *MYD88* L265P mutation had a median of 0.405.

## 4. Discussion

In tumors, functional TLRs expression could influence preferable anti-tumor effects by activating inflammatory cytokine expression and cytotoxic T-cells response. However, an excessive TLRs activation could promote tumor progression, since TLR-induced inflammation stimulates cancer cells boost in the microenvironment [16]. Several reports demonstrated changes of immunological parameters between accumulation and proliferation compartments of the microenvironment in CLL [19,20]. There are some studies that suggest differential signaling trough BCR in an accumulative and proliferative compartment in CLL microenvironment and provide their differential involvement in biology and pathogenesis in this disease [19,21]. It was found that especially in the lymph node microenvironment of CLL, both BCR and TLR signaling could contribute to NF-κB activation, which is essential in immune response regulation, oncogenesis, and tumor progression [21]. So far there are many questions about the expression pattern of TLRs and their prognostic significance in CLL, especially in the bone marrow microenvironment [12,22,23].

Results of our work showed aberrant expression of *TLRs* in CLL patients compared to HVs, proving that *TLRs* expression is differentially regulated in CLL. We showed higher expression of *TLR7* and *TLR9* in CLL compared to HVs, which was confirmed in earlier reports [24,25]. Arvaniti et al. [11] demonstrated high expression of *TLR7*, while the expression of *TLR9* was showed to be low on a mRNA level. Other studies showed variable levels of mRNA and low protein expression of TLR9 [26]. Chatzouli et al. [27] reported that *TLR7* and *TLR9* stimulation with agonists results in apoptosis of CLL cells but only in *IGHV* mutated patients. Recent studies provided by Zhao et al. [28] demonstrated that phenotypically identical cells nevertheless express very different levels of each receptor. The authors suggested that the engagement of multiple receptors, such as TLR7/8/9, may be necessary for improving the host anti-tumor response. Activation of TLR7/8/9 leads to antigen-specific humoral responses through B-lymphocyte activation, but also the inhibition of B-cell apoptosis. In contrast to higher *TLR7* and *TLR9* expression in CLL, expression of *TLR2* and *TLR4* was confirmed to be lower in CLL compared to HVs. Our results are in line with previous studies [7,23,24,25]. The expression of *TLR2* and *TLR7* was correlated with negative known prognostic markers, but only in the bone marrow compartment. Expanded expression analysis of splicing variants of *TLR4* was assessed in CLL patients for the first time. Here, we showed that the expression of splicing variants of *TLR4* (*TLR4(1)* and *TLR4(4))* were significantly lower in PBMC in CLL compared to HV. The decreased expression of splicing variants of *TLR4* observed in this study might then be a result of an impaired host response in CLL patients. Several TLR agonists have been used in clinical trials of CLL patients as adjuvants to improve the efficacy of chemotherapy, for example, the agonist of TLR4. Different studies demonstrate positive as well as negative effects of TLR4 stimulation on cancer development or treatment. Hwang et al. [29] indicated that stimulation of TLR4 results in IL-6, IL-8, IL-12, TNF, INF-γ, and CCL5 secretion. Stimulation of TLR4 that can enhance the anti-tumor response and the beneficial effects of TLR4 stimulation while eliminating the negative effects remains a challenge for cancer researchers.

Signal transduction through TLRs is involved in B cell biology, including activation of naïve B cells, differentiation, and induction proliferation of memory B cells. Expression of TLR is associated with their ability to respond to TLR agonists. Naïve B cells are characterized by low expression of TLR7 and TLR9, as well as TLR1, TLR6, TLR10, while memory B cells express a high level of TLR7 and TLR9, accompanied by a low level of TLR2, TLR4, TLR8 [11,30]. Comparing our defined *TLR* profile of CLL cells to normal B cells, we can indicate that it is parallel to memory B cells. This analogy is in line with the previous study [30].

No differences in TLRs expression between peripheral blood and bone marrow indicate similarities shared between these two distinct compartments of the CLL microenvironment. However, we suggest that differential regulation of *TLRs* accompanies its variable prognostic value, depending on the microenvironment of the specific compartment of CLL. Interestingly, our results for the first time showed the impact of splicing variants of *TLR4(3)* on clinical outcomes in CLL. Analysis of survival curves revealed that TTFT was significantly shorter in the group of patients with a low level of *TLR4(3)* expression compared to the group of patients with a high level of *TLR4(3)* expression in BMMC, thereby we could indicate the negative prognostic value of low *TLR4(3)* expression assessed in the bone marrow compartment in CLL. Moreover, our results might indicate also a potential negative prognostic value of the low level of *TLR4(4)* in bone marrow since expression of TTFT tended to be shorter in the group of patients with a low level of *TLR4(4)* expression compared to the group of patients with a high level of *TLR4(4)* expression in BMMC. Analogous results were not obtained in peripheral blood, as well as in terms of *TLR4(1)*. Thereby, we might indicate that deregulation of TLR4 signaling via *TLR4(3)* and *TLR(4)* is more relevant in bone marrow as a proliferative than accumulative compartment. These differences might be explained by differential posttranscriptional regulation of *TLR4(1)*, *TLR4(3)* and *TLR(4)* and diverse functional and cellular interactions in those two compartments of CLL. Although TLR4 splicing variants differ in terms of the number of exons and length of the extracellular domain, their functional importance in a cell has not been analyzed [31,32]. The significance of TLR4 in disease progression was shown also in human lung cancers [33], as well as ovarian cancer [34]. In mantle cell lymphoma (MCL), Wang et al. [35] showed that signaling through TLR4 triggers a cascade leading to the growth of cells and evasion from immune surveillance, contributing to disease progression. However, Nunez et al. [36] showed that TLR4-activated tumor cells are engaged in an antitumoral immune response. This discrepancy might be associated with the type of cytokines that are secreted upon stimulation in a specific tumor microenvironment.

Results of our study revealed a tendency for shorter TTFT in groups of CLL patients with high *TLR9* expression in comparison to low *TLR9* expression in BBMC, indicating the potential prognostic significance of high mRNA *TLR9* expression in bone marrow in CLL. In PBMC, there was no difference in TTFT when referring to the level of *TLR9* expression, while the results of our previous study [22] revealed that high expression of *TLR9* on the protein level in CLL patients is correlated with longer TTFT. Interestingly, significant discrepancies were identified between mRNA and protein levels for certain TLR expression, and a high expression on a mRNA level did not always correspond to strong protein expression. Several factors could be responsible for these discrepancies, such as cellular intraclonal heterogeneity, differential activation status of malignant cells, and different cell viability in different samples. The expression of *TLR2* and *TLR7* was correlated with known negative prognostic markers but only in the bone marrow compartment.

Since downstream signaling pathways through TLRs involve Myd88 as an adaptor molecule, we provided *MYD88* expression patterns in peripheral blood and bone marrow, referring to CLL prognostic factors as well as an association between *MYD88* and *TLRs* expression in both compartments. In the literature, there is only data about the prognostic significance of *MYD88* mutation [17] or *MYD88* expression. We revealed higher *MYD88* expression in CLL patients compared to HVs, although Antosz et al. [37] revealed lower expression of *MYD88* on a mRNA level in CLL compared to control. They found that TLR agonist stimulation did not result in changes in Myd88 protein expression and suggested some defects in Myd88 proteins in CLL. Myd88 involvement by acting downstream of TLRs in carcinogenesis was shown in many reports concerning cancer of the skin, pancreas, liver, colon, sarcoma [38], whereas the data about the mRNA role of *MYD88* expression in tumors are limited. Chen et al. [39] showed that *MYD88* as well as *TLR4* mRNA expression was higher in breast cancer tissue compared to adjacent normal tissue. Moreover, subsequent research showed a higher protein level of Myd88 and TLR4 in breast carcinoma than paracarcinoma tissue, as well as a correlation between their expression and axillary lymph node metastasis that providing the metastatic potential role of TLR4/Myd88 signaling in breast cancer [40]. However, in ovarian cancer it was also revealed that Myd88 expression strongly correlated with TRL4 expression and provided a favorable prognosis [41].

All TLRs expressions that were analyzed (*TLR2*, *TLR7*, *TLR9,* and *TLR4* isoforms) represent components of downstream cell signaling pathways by Myd88 that finally activate NF-κB, which is essential for CLL survival. It has been proven that TLRs, including 7 and 9, initiate the Myd88-dependent pathway directly, while TLRs types 2 and 4 indirectly impact the involvement of TIRAP. Specifically, TLR4 initiates an alternative pathway which is MyD88-independent by recruiting TRIF that eventually activates NF-κB.

These mutual associations indicate their relevant cooperation which points to the utilization of Myd88 by TLRs signaling in CLL cells. Since the MyD88-dependent signaling pathway leads to the early phase of NF-κB activation whereas the MyD88-independent signaling pathway initiates the late phase of NF-κB activation, this suggests that both mechanisms might be utilized in CLL.

To sum up, our results proved correlations between *MYD88* and analyzed *TLRs* expressions in both compartments, indicating their relevant cooperation in signal transduction in CLL cells. The *MYD88* expression was higher in CLL patients compared to HVs. The TLRs expression was aberrant in CLL patients compared to HVs. Differences in TTFT indicate a negative prognostic value of low *TLR4(3)* expression in the bone marrow, which might suggest an importance of deregulation of the signaling pathway typical for *TLR(4)* (Myd88-independent/TRIF-dependent NF-κB activation) in this compartment.

We proved that *TLRs* expression is differentially regulated in CLL but is similarly shared between two distinct compartments of the CLL microenvironment. We indicate that differential regulation of *TLRs* might accompany the various role of TLRs signaling in peripheral blood and bone marrow, representing two different microenvironmental compartments of CLL.

## Figures and Tables

**Table 1:** Clinical characteristics of chronic lymphocytic leukemia (CLL) patients.

Characteristic	Number of Patients
Sex	
Female	34 (36.2%)
Male	60 (63.8%)
Binet’s classification	
A	38 (40.43%)
B	39 (41.49%)
C	14 (14.89%)
ZAP-70 (cut off 20%)	
Positive	28 (29.8%)
Negative	58 (61.7%)
NA (not available)	8 (8.5%)
CD38 (cut off 30%)	
Positive	18 (19.2%)
Negative	68 (8.5%)
NA (not available)	8 (72.3%)
Mutational status of the immunoglobulin heavy-chain variable-region (*IGHV)*	
Mutated	28(29.8%)
Unmutated	41 (43.6%)
NA (not available)	25 (26.6%)

**Table 2:** Primer sequences for the examined toll-like receptors (TLRs).

Type of TLR (Splicing Variants)	Sequences of Primers
TLR2	F: 5′ CAA GCA GGA TCC AAA GGA GA 3′ R: 5′ ACC CAC ACC ATC CAC AAA GT 3′
TLR4 (1)	F: 5′GGC TCG AGG AAG AGA AGA CA 3′ R: 5′ATT AGG AAC CAC CTC CAC GC 3′
TLR4 (3)	F: 5′CTG TGG GGC GGC TCG AGG AA 3′ R: 5′GCC AAG TCT CCA CGC AGG GCT 3′
TLR4 (4)	F: 5′CGG TGA TAG CGA GCC ACG CA 3′ R: 5′GGA TTT CAC ACC TCC ACG CAG GG 3′
TLR7	F: 5′ ATC ACT CCA TGC CAT CAA GA 3′ R: 5′ CCC CAA GGA GTT TGG AAA TTA 3′
TLR9	F: 5′ GCC AGA CCC TCT GGA GAA 3′ R: 5′ AGA CTT CAG GAA CAG CCA GTT G 3′

F, forward, R, reverse.

**Table 3:** Correlations between *TLR* and *MYD88* expression in peripheral blood mononuclear cells (PBMC) and bone marrow mononuclear cells (BMMC) of CLL patients.

	PBMC			BMMC		
	95% CI	r	*p*	95% CI	r	*p*
*TLR2* *MYD88*	0.112–4.919 0.000–0.634	0.722	<0.0001	0.106–0.612 0.110–0.335	0.388	0.0001
*TLR4(1)* *MYD88*	0.078–0.332 0.115–0.634	0.599	<0.001	0.080–0.341 0.110–0.335	0.284	0.007
*TLR4(3)* *MYD88*	0.095–0.685 0.115–0.634	0.558	<0.001	0.078–0.471 0.110–0.335	0.299	0.005
*TLR4(4)* *MYD88*	0.104–1.271 0.115–0.634	0.560	<0.001	0.063–0.654 0.110–0.335	0.440	<0.001
*TLR7* *MYD88*	0.066–6.382 0.000–0.398	0.580	<0.001	0.137–11.641 0.110–0.335	0.358	<0.001
*TLR9* *MYD88*	0.096–2.392 0.000–0.497	0.492	<0.001	0.166–7.692 0.110–0.294	0.541	<0.001

## Data Availability

The data presented in this study are available on request from the corresponding author.

## Supplementary Materials

The following are available online at https://www.mdpi.com/2077-0383/10/4/867/s1, Figure S1: 637 Correlations between *TLR2* expression and *MYD88* expression in PBMC (A) and BMMC (B) in CLL., Figure S2: 638 Correlations between *TLR4* splice variants expression and *MYD88* expression in PBMC (A, C, E), and BMMC (B,D,F) in CLL., Figure S3: 639 Correlations between *TLR7* expression and *MYD88* expression in PBMC (A) and BMMC (B) in CLL., Figure S4: 640 Correlations between *TLR9* expression and *MYD88* expression in PBMC (A) and BMMC (B) in CLL., Table S1. Expression of *TLRs* in A, B, C clinical stage of disease according to Binet scale.
