# Supplementary material for: Aberrant Expression of TLR2, TLR7, TLR9, Splicing Variants of TLR4 and MYD88 in Chronic Lymphocytic Leukemia Patients
Source: J Clin Med. 2021 Feb 19;10(4):867. doi: 10.3390/jcm10040867 (PMC7922273; doi:10.3390/jcm10040867)
Supplement: Supplementary file 1 [file jcm-10-00867-s001.pdf]

Supplementary Materials

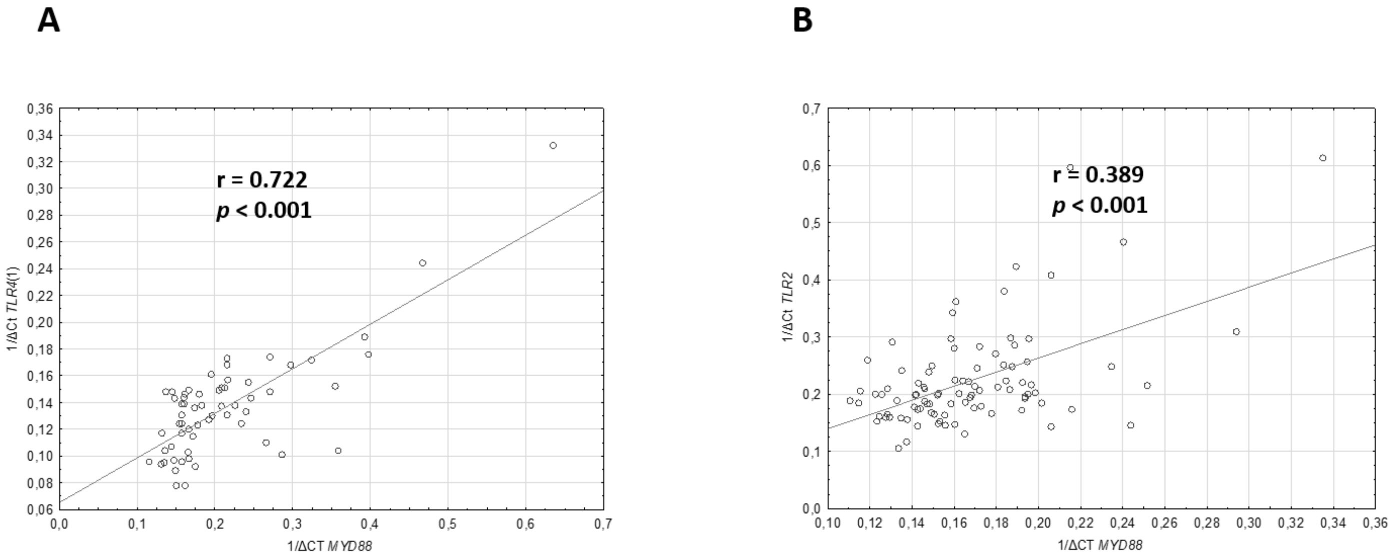

Figure S1. 637 Correlations between *TLR2* expression and *MYD88* expression in PBMC (**A**) and BMMC (**B**) in CLL.

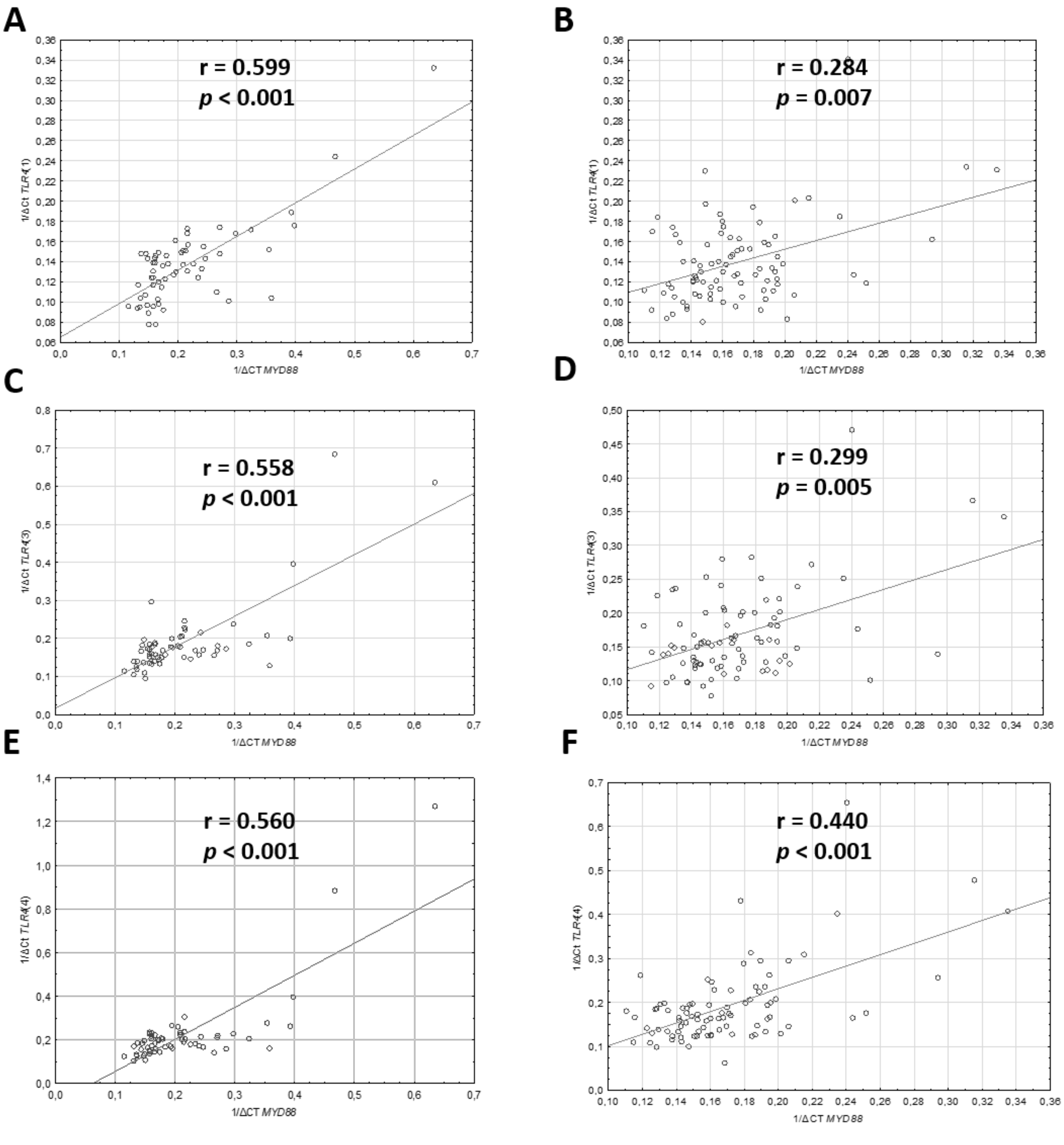

Figure S2. 638 Correlations between *TLR4* splice variants expression and *MYD88* expression in PBMC (**A**, **C**, **E**), and BMMC (**B**,**D**,**F**) in CLL.

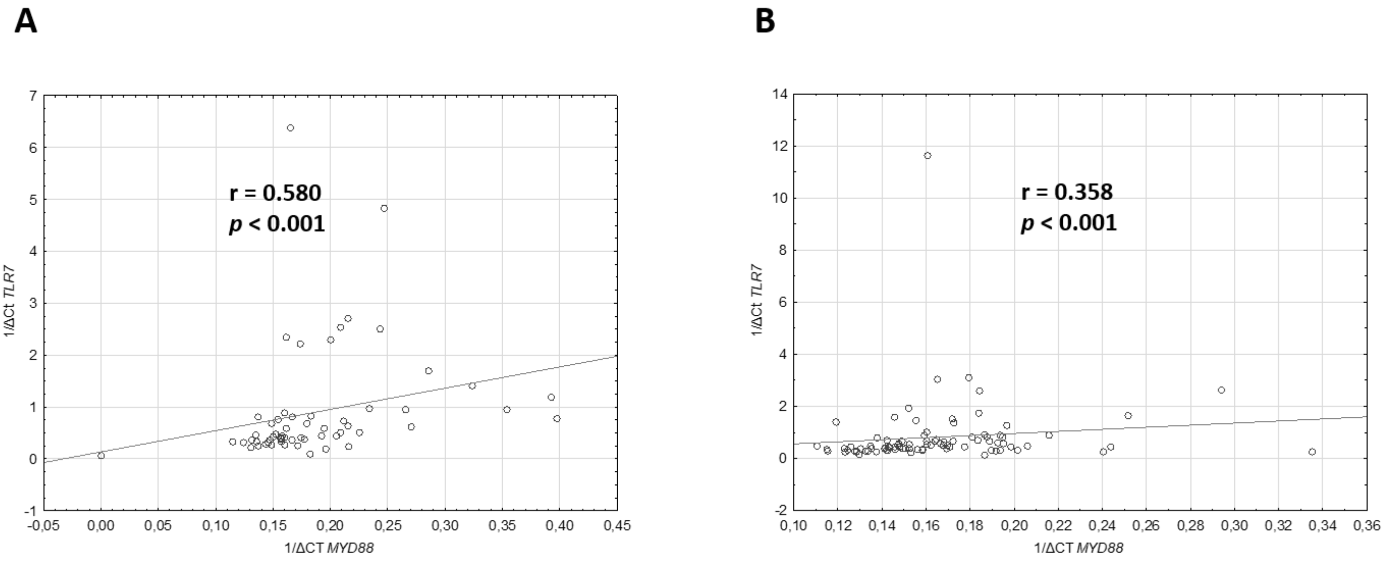

Figure S3. 639 Correlations between *TLR7* expression and *MYD88* expression in PBMC (**A**) and BMMC (**B**) in CLL.

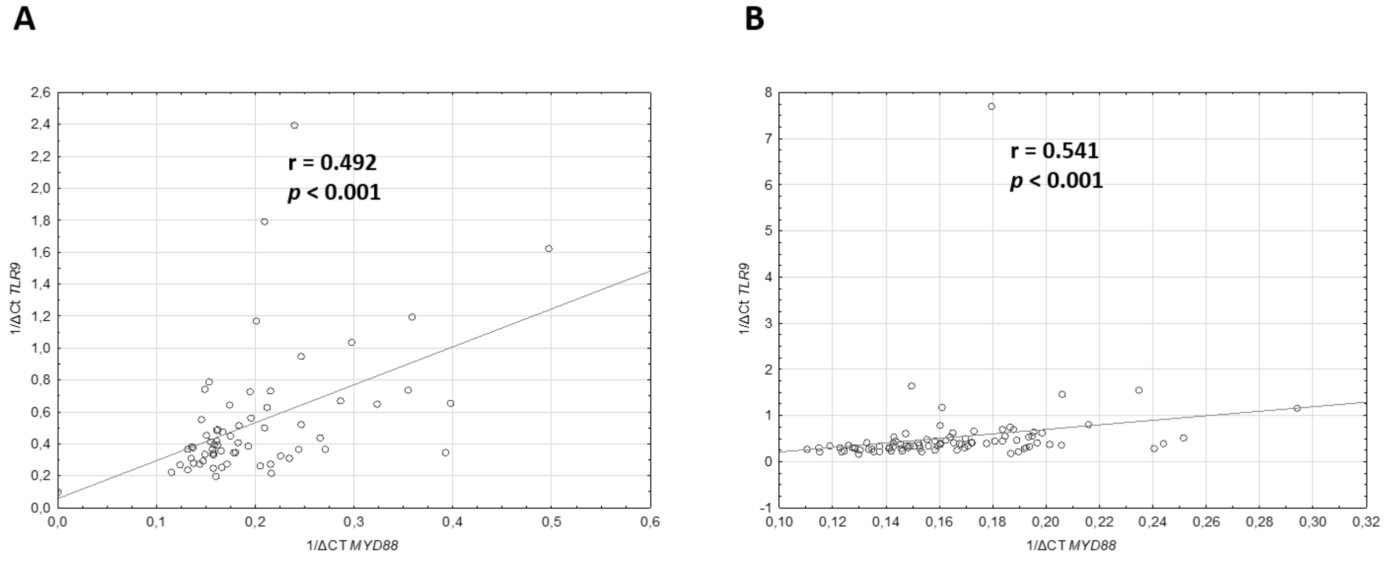

Figure S4. 640 Correlations between *TLR9* expression and *MYD88* expression in PBMC (**A**) and BMMC (**B**) in CLL.

Table S1. Expression of *TLR*s in A, B, C clinical stage of disease according to Binet scale.

**Binet`s stage** ***TLR***	**A**	**B**	**C**	**Statistical significance (*p*)**
TLR2 (PBMC)	0.225	0.1995	0.263	0.068
TLR2 (BMMC)	0.2010	0.1995	0.2070	0.96
TLR4(1) (PBMC)	0.1340	0.1360	0.1430	0.94
TLR4(1) (BMMC)	0.130	0.1270	0.1380	0.7123
TLR4(3) (PBMC)	0.1755	0.1570	0.1660	0.5377
TLR4(3) (BMMC)	0.1575	0.1520	0.1390	0.4459
TLR4(4) (PBMC)	0.2055	0.1830	0.1670	0.4907
TLR4(4) (BMMC)	0.1705	0.1760	0.1530	0.9505
TLR7 (PBMC)	0.4430	0.6415	0.4510	0.2273
TLR7 (BMMC)	0.4980	0.4365	0.3950	0.9978
TLR9 (PBMC)	0.3900	0.3660	0.5330	0.3733
TLR9 (BMMC)	0.3870	0.4135	0.290	0.1216

PBMC- peripheral blood mononuclear cells, BBMC- bone marrow mononuclear cells.
